# Master control: transcriptional regulation of mammalian *Myod*

**DOI:** 10.1007/s10974-019-09538-6

**Published:** 2019-07-12

**Authors:** Fiona C. Wardle

**Affiliations:** Randall Centre for Cell & Molecular Biophysics, King’s College London, New Hunt’s House, Guy’s Campus, London, SE1 1UL UK

**Keywords:** Myod, Myogenesis, Mammalian embryo, Transcription factor, Epigenetic regulation

## Abstract

MYOD is a master regulator of the skeletal myogenic program. But what regulates expression of *Myod*? More than 20 years ago, studies established that *Myod* expression is largely controlled by just two enhancer regions located within a region 24 kb upstream of the transcription start site in mammals, which regulate *Myod* expression in the embryo, fetus and adult. Despite this apparently simple arrangement, *Myod* regulation is complex, with different combinations of transcription factors acting on these enhancers in different muscle progenitor cells and phases of differentiation. A range of epigenetic modifications in the *Myod* upstream region also play a part in activating and repressing *Myod* expression during development and regeneration. Here the evidence for this binding at *Myod* control regions is summarized, giving an overview of our current understanding of *Myod* expression regulation in mammals.

## Introduction

*Myogenic determination gene number 1* (*Myod1*, usually simply referred to as *Myod*) was isolated on account of to its ability to induce skeletal muscle differentiation in fibroblasts, and a variety of other non-skeletal muscle cells (Choi et al. 1990; Davis et al. 1987; Weintraub et al. 1989). Due to this ability to reprogramme other cells to muscle and because it is expressed in cells of the early muscle lineage, *Myod* is often referred to as a master regulator (Chan and Kyba 2013; Weintraub et al. 1989), although it should be noted that *Myod* expression is not sufficient to reprogramme all non-muscle cells (e.g. Albini et al. 2013).

*Myod* encodes a basic helix-loop-helix transcription factor belonging to a larger family of myogenic regulatory factors (MRFs) that together control determination and differentiation of all skeletal muscle cells. *Myogenic Factor 5* (*Myf5*), *Myogenic Regulatory Factor 4* (*Mrf4*, also known as *Myf6*) and *Myod* act to direct cells into the skeletal myogenic lineage (Kassar-Duchossoy et al. 2004; Rudnicki et al. 1993). In the absence of all three factors myoblasts, and consequently skeletal muscles, do not form in the embryo (Kassar-Duchossoy et al. 2004; Rudnicki et al. 1993). While *Myod* and *Myf5* can functionally substitute for each other to maintain myogenesis during development, in the absence of *Myf5* and *Myod*, *Mrf4* cannot drive fetal skeletal muscle differentiation (Kassar-Duchossoy et al. 2004). Expression of a fourth MRF, *Myogenin* (*Myog*), which is required for terminal differentiation of skeletal muscle cells in the embryo, is induced by the other three MRFs (Hasty et al. 1993; Nabeshima et al. 1993). Likewise, the MRFs regulate myogenesis in satellite cells, a stem cell population that mediates muscle homeostasis, growth and repair in adult muscle (reviewed in Zammit 2017).

Since MRFs act to drive myogenesis, their expression must be under strict spatiotemporal control during development and regeneration. In the case of *Myod*, studies dating back over two decades have shown that its expression in the embryo and adult is controlled by two cis-regulatory elements located upstream of the transcription start site (TSS) together with a minimal promoter region (Asakura et al. 1995; Chen et al. 2001, 2002; Faerman et al. 1995; Goldhamer et al. 1992, 1995; L’Honore et al. 2003; Tapscott et al. 1992). These elements bind both activators and repressors of transcription in different combinations depending on the cell type. The locus is also subject to epigenetic regulation, with modification of histones, DNA methylation and interaction with noncoding RNA all contributing to the correct regulation of expression. This review summarises the factors that have been shown to bind upstream of *Myod* and the evidence that they are involved in regulation of *Myod* expression in mammals.

## Skeletal muscle development and regeneration

The muscles of the limbs and trunk are derived from somites, epithelial structures that bud off in a rostral-caudal sequence from the paraxial mesoderm (reviewed in Tajbakhsh and Buckingham 2000). In mouse, the first somite pair forms at around embryonic day 8 (E8) and there after a new pair of somites forms caudally every 2 h or so until approximately 65 somites are formed (Forsberg et al. 1998; Tam 1981). The dorsal part of the somite forms the dermomyotome (DM), from which the dermis and skeletal muscle of the trunk and limbs will develop. Soon after the somite forms, progenitor cells at the edges (lips) of the DM migrate under the DM and form the myotome (Venters et al. 1999). Subsequently, the DM begins to lose its epithelial character and cells from the central portion of the DM also move into the myotome, contributing further to its growth (Relaix et al. 2005). Cells of the dorsomedial myotome will form the epaxial (back) muscles while those in the ventrolateral region will form hypaxial muscles—the diaphragm, limb and ventral body wall muscles. At the level of the limb buds, cells in ventrolateral lip of the DM migrate into the limbs, where they then proliferate and differentiate (Houzelstein et al. 1999). Muscles of the head are primarily derived from the cranial mesoderm, which comprises cells originating from the anterior lateral plate, unsegmented paraxial mesoderm (rostral to the somite forming paraxial mesoderm) and prechordal mesoderm, although some neck, jaw and tongue muscle progenitors migrate to the head from the most rostral (occipital) somites (Michailovici et al. 2015).

This wave of primary myogenesis is complete by approximately E14.5 in mouse and is closely followed by a second, fetal, wave of myogenesis, where myoblasts proliferate and fuse with pre-existing primary myofibres or form de novo myofibres through fusion with each other. During this phase, which lasts until birth, some cells remain undifferentiated, forming a pool of progenitors, or satellite cells, which become located under the basal lamina surrounding each myofibre. These satellite cells are quiescent in adult muscle, but can be induced to proliferate and differentiate in response to various stimuli, including exercise and injury (Relaix and Zammit 2012).

The first myogenic genes to be transcribed are *Myf5* and *Mrf4*, in the DM and then the myotome, shortly followed by branchial arch expression. *Myf5* expression is also activated in the limb muscle precursors following their migration to the limb (reviewed in Comai and Tajbakhsh 2014; Vicente-García and Carvajal 2018). Expression of *Myod* follows *Myf5* and continues in muscle cells until birth when expression is downregulated, although a low level of *Myod* transcription endures in certain adult skeletal muscles. Expression of *Myod* is also reactivated in activated satellite cells during muscle regeneration.

## Other models of myogenesis

In addition to in vivo investigation, many studies of myogenic development make use of cell lines that are easily differentiated to myotubes or which express high levels of MRFs. In particular, C2 cell line clones, such as C2C12 and C2.7, undergo proliferation in high serum growth conditions, but differentiate and form contractile myofibres when serum is limited. These cells most likely represent a model of satellite cell differentiation, since they are derived from injured adult mouse muscle (Yaffe and Saxel 1977). In addition, Rhabdomyosarcoma cells are characterized by expression of MRFs, including *Myod* (Clark et al. 1991), and are a model for understanding regulation of MRFs (Taberlay et al. 2011).

## Cis-regulatory elements controlling *Myod* expression

Gene expression is regulated, in part, by enhancers, genomic regulatory elements that bind transcription factors, interact with target promoters, recruit RNA Polymerase II (RNAPII) and promote transcription. Since enhancers are functional even outside their endogenous context, reporter assays are often used to test their ability to activate gene expression in cell culture or transgenic animals.

**Fig. 1:**
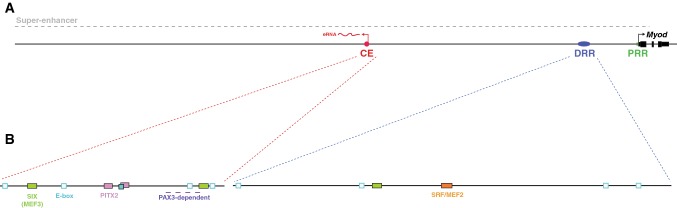
Sequences that regulate *Myod* expression. **a** Schematic of a region 50 kb upstream of the *Myod* gene, including the core enhancer (CE, in red), the distal regulatory region (DRR, in blue) and the proximal regulatory region (PRR, in green) immediately upstream of the *Myod* transcription start site (TSS, black arrow). This region lies within a super enhancer, associated with high levels of transcription factors occupancy, H3K27ac marks and multiple enhancer RNAs (eRNAs). One of these is an eRNA is transcribed from the CE that plays a role in regulating *Myod* expression through promoting chromatin accessibility at the *Myod* promoter. **b** Transcription factor binding sites for bHLH factors (E-box) including MYOD, MYF5, CLOCK, BMAL2, SIM2, MSC and TCF21 are shown in blue, for SIX and EYA factors in green (MEF3 site), for PITX2 in pink (Paired binding site) and for SRF/MEF2 in orange (CaRG box). Closed boxes show sites that have been shown to bind these factors in electromobility shift or other assays are shown as closed boxes, while open boxes show predicted sites. The E-box (closed) that binds CLOCK/BMAL2 overlaps a PIXT2 binding site. A region that is PAX3 dependent in somites and limbs, and is required for expression in myotomally-derived muscles, is indicated by the dashed purple line. (Color figure online)

A series of experiments using reporter assays in vitro and in vivo showed that at a region extending 24 kb upstream of the human *MYOD* gene can essentially recapitulate the endogenous *Myod* expression pattern in the mouse embryo, fetus, and adult muscle, including regenerating satellite cells. Remarkably, a large amount of this expression is controlled by just two enhancers, which have come to be known as the Core Enhancer (CE) and the Distal Regulatory Region (DRR), together with a proximal regulatory region (PRR) immediately upstream of the transcription start site (TSS; Fig. 1a; Asakura et al. 1995; Chen et al. 2001, 2002; Faerman et al. 1995; Goldhamer et al. 1992, 1995; L’Honore et al. 2003; Tapscott et al. 1992).

The CE, which was first identified as a 258 bp region located 20 kb upstream of the TSS of the human *MYOD* gene, shows 89% sequence identity with a similar region 23 kb upstream of the mouse *Myod* gene (Fig. 1a; Goldhamer et al. 1995). The CE drives initiation of *Myod* expression in muscle precursor cells and nascent myocytes of the DM and myotome, branchial arches and limb buds in a pattern that mirrors the endogenous gene (Chen et al. 2001; Goldhamer et al. 1995). However, the CE is not sufficient for continued maintenance of reporter gene expression in differentiated skeletal muscle, since the transgene activity decreases during fetal and postnatal development until it becomes largely undetectable in adult muscle (Faerman et al. 1995). Linker-scan mutagenesis of the CE reveals elements within it that are essential for expression in all skeletal muscle progenitors, including the head, and separate elements that are required only for expression in the epaxial muscles and the hypaxial muscles of the body wall (Kucharczuk et al. 1999).

Although sufficient to drive early expression, deletion of the CE within the context of the 24 kb *MYOD r*eporter construct results merely in reduced expression of the reporter in the hypaxial myotome together with a delay in expression in branchial arches and limb buds (Chen et al. 2001), indicating other regions in the 24 kb construct can compensate in part for the loss of the CE. Indeed, deletion of the CE within the mouse genome shows the CE is not absolutely required to initiate endogenous *Myod* expression in somites, since this does not change timing or pattern of expression, although it does lead to weaker expression of *Myod*. However, initiation of *Myod* expression in branchial arch and limb buds is delayed, confirming the CE controls the timely initiation of *Myod* expression in these muscles (Chen and Goldhamer 2004).

The DRR is a 714 bp region located approximately 5 kb upstream of mouse *Myod*, and also shares high sequence conservation with its human counterpart (Fig. 1a; Chen et al. 2001; Tapscott et al. 1992). In transgenic mouse reporter studies, the DRR drives expression at a later stage than the CE, in differentiated skeletal muscle cells of the myotome, head and limb (Asakura et al. 1995; Chen et al. 2001; Kablar et al. 1997). In addition, it continues to drive reporter expression in adult skeletal muscle cells in a pattern that resembles endogenous *Myod* expression (Hughes et al. 1993), and upregulation in activated satellite cells in regenerating muscle (L’Honore et al. 2003). Deletion of the DRR in the mouse genome causes a reduction of early *Myod* expression before cells differentiate, while later expression of *Myod* in the embryo and fetus is unaffected, results that are unexpected in light of the transgenic studies showing it drives expression in differentiated cells, and suggesting redundancy with other sequences in the *Myod* locus at later stages. However, the DRR is required to maintain levels of *Myod* expression in adult skeletal muscle, consistent with transgenic assays (Chen et al. 2002). Taken together these studies indicate that in general the CE is required for initiation of *Myod* expression and the DRR with maintenance of expression and adult skeletal muscle expression, but that redundancy with each other and other regions exists.

The PRR, which extends 275 bp upstream of the *Myod* TTS, is unable to direct specific expression of *Myod*, but acts as a minimal promoter in combination with the DRR or CE (Fig. 1a).

## Transcription factor binding to *Myod* cis-regulatory elements: activators

Despite a role for *Myod* in all skeletal muscle groups, genetic studies show that the factors acting upstream to regulate expression are varied and differ depending on the origin of the cells and the phase of muscle formation (reviewed in Buckingham 2017). Summarised below, for those factors that are implicated in direct regulation of *Myod* expression, is evidence for binding of these factors to the CE and DRR, and other regions within in the proximal upstream region of *Myod.*

### MRFs and PAX3/PAX7

In the somites and limbs, *Pax3* and *Myf5* act in parallel pathways to regulate *Myod* expression during the first wave of myogenesis (Tajbakhsh et al. 1997). *Pax3* expression precedes *Myod* in the DM and cells that migrate to the limb. *Pax7* becomes expressed a little later in the central DM and plays a redundant role in embryonic myogenesis with *Pax3*. Indeed, although the early myotome forms in *Pax3*; *Pax7* mutant mice, no further muscle forms after this (Relaix et al. 2005). Consistent with this, *Myod* expression is almost completely absent in *Pax3*/*Pax7* double mutant embryos at later embryonic stages (E13.5; Relaix et al. 2005).

**Fig. 2:**
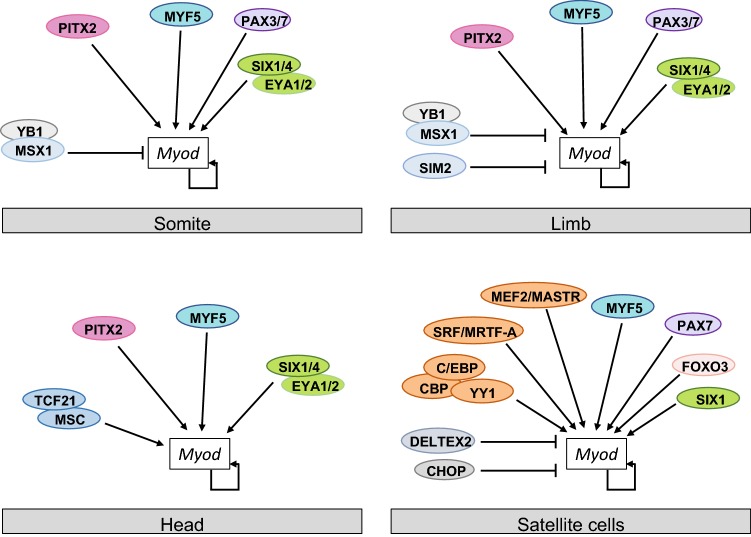
Transcription factor binding and regulation of *Myod* in different cell types. Factors that regulate *Myod* expression and have been shown to bind the CE, PRR, DRR or adjacent regions are shown for different cells types. See text and Table 1 for details

As mentioned above, *Myf5* and *Mrf4* expression also precedes *Myod* expression. While loss of *Myf5* alone does not impact *Myod* expression, loss of *Myf5* and *Mrf4*, in a mutant where targeted disruption of the *Myf5* locus also results in lack of *Mrf4* expression due to the genes being closely adjacent to each other (henceforth referred to as *Myf5*(*Mrf4*) mutants), causes an approximately 2-day delay in expression of *Myod* in the somites (Kablar et al. 1997; Kassar-Duchossoy et al. 2004; Tajbakhsh et al. 1997). Similarly, loss of Pax3 alone does not affect *Myod* expression, whereas *Pax3*;*Myf5*(*Mrf4)* mutants do not express *Myod* in somites and limb, indicating these factors compensate for each other in regulating *Myod* in the trunk of the embryo (Kassar-Duchossoy et al. 2004; Fig. 2). Craniofacial *Myod* expression on the other hand is seen in these mutants, since *Pax3* is not expressed in cranial mesoderm, although Pax7 is (Tajbakhsh et al. 1997; Fig. 2). Instead, PITX2 and other transcription factors play a role in regulating *Myod* expression in the head mesoderm (see sections below).

A subset of *Pax3/Pax7* expressing cells from the embryonic somites will become adult satellite cells and continue to express Pax7, which is required for satellite cell function in muscle homeostasis and regeneration (Kuang et al. 2006; Oustanina et al. 2004; Relaix et al. 2006; von Maltzahn et al. 2013). Pax7 is able to activate the expression of *Myod*, and after satellite cell activation both *Myf5* and *Myod* are required for normal muscle regeneration, since when *Myf5* and *Myod* are together knocked out in adult muscle, satellite cells fail to reliably enter the myogenic pathway and do not differentiate (Gayraud-Morel et al. 2007; Megeney et al. 1996; Ustanina et al. 2007; Yamamoto et al. 2018; Zammit et al. 2006).

Linker-scan mutagenesis of the CE suggests a region near the 3′ end (LS14 and 15, Kucharczuk et al. 1999) is dependent on PAX3 activity in the somites and limbs (Fig. 1b), however, direct binding of PAX3 (or PAX7) had not been shown at the CE, or indeed the DRR, which may indicate these regions are indirectly regulated by PAX3/PAX7. However, several cell-based studies have shown binding of PAX/PAX7 to *Myod* upstream regions other than the CE or DRR. In myoblasts transfected with tagged PAX3 or PAX7, PAX7 was shown by chromatin immunoprecipitation (ChIP) to bind approximately 40 kb upstream of the TSS (Fig. 2; Table 1; Soleimani et al. 2012a). Other experiments using an antibody which recognises both PAX3 and PAX7 showed that in partnership with FOXO3, PAX3/PAX7 binds a region between − 1.5 and − 1.6 kb upstream of the *Myod* TSS to synergistically regulate *Myod* expression (Table 1; Fig. 2; Hu et al. 2008). This binding is associated with increased recruitment of RNAPII to the *Myod* promoter (Hu et al. 2008). FOXO3 is further implicated as a regulator of endogenous *Myod* expression, since in mice mutant for *Foxo3, Myod* is down-regulated in regenerating muscle (Fig. 2; Hu et al. 2008).

**Table 1:** Protein binding identified at the *Myod* proximal regulatory region (PRR), distal regulatory region (DRR), core enhancer (CE) and adjacent regions

Region	Interacting protein	Binding shown by	Binding site^a^	References
PRR	MSC	ChIP-qPCR in mouse embryo branchial arch tissue	Not characterised	Moncaut et al. (2012)
	TCF21	ChIP-qPCR in mouse embryo branchial arch tissue	Not characterised.	Moncaut et al. (2012)
	RAD21	ChIP-qPCR in Rhabdomyosarcoma cells	Not characterised	Taberlay et al. (2011)
	p300	ChIP-qPCR in C2C12 cells	Not characterised	Hamed et al. (2013)
	MYOD	ChIP-seq in C2C12 cells and primary myoblasts	Not characterised	Mousavi et al. (2013) and Umansky et al. (2015)
	MYF5	ChIP-seq in mouse embryonic fibroblasts transduced with MYF5	Not characterised	Conerly et al. (2016)
	PITX2	ChIP-PCR in C2C12 cells and extraocular eye muscle cell line	Not characterised	Zacharias et al. (2011)
	DELTEX2	ChIP-qPCR in C2C12 cells	Not characterised	Luo et al. (2017)
Inter PRR–DRR	PITX2	ChIP-qPCR in hindlimb buds and myotome. EMSA with in vitro translated PITX2	PITX2 binding site at − 615 bp	L’Honore et al. (2010)
	MYOD	EMSA with nuclear extracts from hindlimb muscle, G8 myoblasts and C3H/lOT1/2 fibroblasts	Two E boxes at − 1750 and − 865 bp	Zingg et al. (1994)
	GLI2	ChIP-pPCR in P19 cells	GLI consensus binding sites at − 1.6 kb and − 0.6 kb	Voronova et al. (2013)
	FOXO3	ChIP-PCR antibody in C2C12 cells. EMSA with recombinant FOXO3	Two Forkhead Regulatory Elements at − 940 bp and − 1598 bp	Hu et al. (2008)
	PAX3/7	ChIP-PCR in C2C12 cells. EMSA with recombinant PAX3 and PAX7	Paired binding site at − 1502 bp	Hu et al. (2008)
	CHOP	ChIP-PCR with C2C12 cells	Not characterised. Primers bind at − 3 kb and − 4 kb	Alter and Bengal (2011)
DRR	SIX1/4	ChIP-seq and -qPCR in C2C12 cells and satellite cell derived myoblasts. EMSA with in vitro translated SIX1 and SIX4	MEF3 site in DRR	Le Grand et al. (2012), Liu et al. (2010) and Relaix et al. (2013)
	EYA1/2	ChIP-qPCR in mouse embryo trunk muscle precursor cells	Not characterised, likely to be via SIX binding at MEF3 site	Relaix et al. (2013)
	PITX2	ChIP-qPCR in mouse embryo myotome	Not characterised	L’Honore et al. (2010)
	MSC	ChIP-qPCR in mouse embryo branchial arch tissue	Not characterised	Moncaut et al. (2012)
	TCF21	ChIP-qPCR in mouse embryo branchial arch tissue	Not characterised	Moncaut et al. (2012)
	SRF	ChIP-qPCR in C2C12 cells. EMSA with nuclear extracts from C2.7 cells	Divergent CaRG box (a hybrid SRF/MEF2 binding site)	L’Honore et al. (2003) and L’Honore et al. (2007)
	MEF2	ChIP-qPCR in C2C12 cells.. EMSA with nuclear extracts from C2C12 cells	Divergent CaRG box (a hybrid SRF/MEF2 binding site), and a canonical MEF2 binding site	L’Honore et al. (2007) and Mokalled et al. (2012)
	YY1	EMSA with nuclear extracts from C2.7 cells	Divergent CaRG box (a hybrid SRF/MEF2 binding site)	L’Honore et al. (2003)
	MRTF-A	ChIP-qPCR in C2C12 cells	Divergent CaRG box (a hybrid SRF/MEF2 binding site)	Mokalled et al. (2012)
	MASTR	ChIP-qPCR in C2C12 cells	Divergent CaRG box (a hybrid SRF/MEF2 binding site), and a canonical MEF2 binding site	Mokalled et al. (2012)
	C/EBP	EMSA with nuclear extracts from C2.7 cells	Divergent CaRG box (a hybrid SRF/MEF2 binding site)	L’Honore et al. (2003)
	p300	EMSA with nuclear extracts from C2.7 cells. ChIP-qPCR in C2C12 cells	Divergent CaRG box (a hybrid SRF/MEF2 binding site)	L’Honore et al. (2003) and Hamed et al. (2013)
	MYOD	ChIP-seq in C2C12 cells and primary myoblasts.	Not characterised	Mousavi et al. (2013) and Umansky et al. (2015)
	MYF5	ChIP-seq in mouse embryonic fibroblasts transduced with MYF5	Not characterised	Conerly et al. (2016)
	DELTEX2	ChIP-qPCR in C2C12 cells.	Not characterised	Luo et al. (2017)
Inter DRR–CE	MYOD	ChIP-seq in C2C12 cells and primary myoblasts	Not characterised. Chip-seq peaks map to region around − 10.5 kb and − 12 kb	Conerly et al. (2016), Cui et al. (2017) and Umansky et al. (2015)
	MYF5	ChIP-seq in mouse embryonic fibroblasts transduced with MYF5	Not characterised. Chip-seq peaks map to region around − 10.5 kb	Conerly et al. (2016)
CE	SIX1/4	ChIP -seq and -qPCR in C2C12 cells. EMSA with in vitro translated SIX1 and SIX4	Two MEF3 sites	Liu et al. (2010) and Relaix et al. (2013)
	EYA1/2	ChIP-qPCR in mouse embryo trunk muscle precursor cells	Not characterised, likely to be via SIX binding at MEF3 site	Relaix et al. (2013)
	PITX2	ChIP-qPCR in hindlimb buds and myotome. EMSA with in vitro translated PITX2	Two PITX2 binding sites	L’Honore et al. (2010)
	RAD21	ChIP-qPCR in Rhabdomyosarcoma cells	Not characterised	Taberlay et al. (2011)
	LSD1	ChIP-qPCR antibody in C2C12 cells	Not characterised	Scionti et al. (2017)
	CLOCK	ChIP-PCR in muscles from mice kept in a 12 h light–dark cycle. EMSA with extract from C2C12 cells overexpressing CLOCK and BMAL	Divergent E-box (CAGCTT)	Andrews et al. (2010) and Zhang et al. (2012)
	BMAL2	ChIP-PCR in muscles from mice kept in a 12 h light–dark cycle. EMSA with extract from C2C12 cells overexpressing CLOCK and BMAL	Divergent E-box (CAGCTT)	Andrews et al. (2010) and Zhang et al. (2012)
	MYOD	ChIP-seq in mouse embryonic fibroblasts transduced with Myod, in C2C12 cells, and primary myoblasts	Not characterised	Conerly et al. (2016), Cui et al. (2017), Mousavi et al. (2013) and Umansky et al. (2015)
	MYF5	ChIP-seq with in mouse embryonic fibroblasts transduced with MYF5	Not characterised	Conerly et al. (2016)
	YB1	ChIP-PCR and -qPCR in C2C12 cells expressing	Not characterised	Lee et al. (2004) and Song and Lee (2010)
	MSX1	ChIP-PCR in C2C12 cells and mouse embryo limbs	Not characterised	Lee et al. (2004), Song and Lee (2010) and Wang et al. (2011)
	SIM2	ChIP-PCR in mouse embryo limb buds	Not characterised	Havis et al. (2012)
Upstream of CE	GLI2	ChIP-pPCR in P19 cells overexpressing GLI2	GLI consensus binding sites at − 32 kb and − 36 kb	Voronova et al. (2013)
	PAX7	Chromatin Tandem Affinity Purification (ChTAP) in satellite cell-derived myoblasts	Not characterised. Chip-seq peaks map to region around − 40 kb	Soleimani et al. (2012b)

^a^Characterized binding sites are those sites where physical binding and/or function had been validated via EMSA and/or luciferase assays, including mutation of the binding site. For some ChIP-seq studies binding was determined through analysis of files downloaded from the Gene Expression Omnibus (Conerly et al. 2016; GEO accession: GSE75370; Cui et al. 2017; GEO accession: GSE63716; Umansky et al. 2015, GEO accession: GSE56131)

MYF5, MRF4, and MYOD itself, regulate the expression of *Myod* through the DRR, since the DRR cannot drive reporter gene expression in *Myod*;*Myf5(Mrf4)* mutant embryos (Kablar et al. 1999). In these embryos the CE drives weak expression in the somites and delayed expression in the limb (Kablar et al. 1999). MYOD can also regulate its own expression in vitro via binding E-boxes, binding sites for bHLH factors, in the promoter region of *Myod* (Fig. 2; Table 1; Zingg et al. 1994). Although the DRR and CE contain E-boxes, direct binding of MRFs has not been shown as yet in embryos and whether these factors differentially bind to the CE and DRR is not known. However, several genome-wide studies have shown MYOD and MYF5 bind the CE and/or the DRR and adjacent sites in myoblasts in culture, although occupancy at these sites differs depending on the cell type and specific assay, so how this binding translates to functional regulation is still to be investigated (Table 1; Conerly et al. 2016; Cui et al. 2017; Mousavi et al. 2013; Soleimani et al. 2012b; Umansky et al. 2015).

### SIX and EYA

Six homeodomain (SIX) transcription factors and their co-factors, members of the Eyes absent (EYA) family, are also upstream regulators of *Myod* in both trunk and head skeletal muscle. Mice mutant for *Six1/4* or *Eya1/2* show a reduction in *Myod* expression in muscle precursor cells of the somite and limb (Grifone et al. 2007; Laclef et al. 2003). Both the CE and DRR bind SIX1/SIX4 and SIX2 in vitro and EYA proteins in vivo (Table 1; Fig. 2; Liu et al. 2010; Relaix et al. 2013), and consistent with a direct role in regulating the CE, when SIX binding sites in the CE are mutated, the CE is unable to drive robust reporter expression in the trunk (Relaix et al. 2013). Despite expression of *Myod* in the head in *Six1/4* and *Eya1/2* mutants, mutation of the SIX binding sites in the CE results in loss of reporter expression in the head. This suggests another SIX factor, likely to be SIX2, activates expression of *Myod* in the head in the absence of *Six1* and *Six4* (Fig. 2; Relaix et al. 2013). SIX1 also appears to regulate *Myod* expression in regenerating adult muscles cells, since SIX1 is bound to the DRR in differentiating satellite cells derived from adult mouse, and when SIX1 activity is knocked down in these cells *Myod* expression is reduced (Table 1; Fig. 2; Le Grand et al. 2012).

### PITX2

PITX2, a paired-like homeodomain protein, is another factor that regulates *Myod* expression in both the trunk and head (Diehl et al. 2006; Dong et al. 2006; L’Honore et al. 2010). In the limb and myotome PITX2 cooperates with MYF5 and MYF4 to regulate *Myod* expression, while in the extraocular eye muscles of the head it acts upstream of *Myf5* and *Mrf4* (reviewed in Hernandez-Torres et al. 2017). PITX2 binds the CE (but not the DRR) in hindlimb buds and is able to activate expression of a reporter gene in cell based assays, while mutation of two paired binding sites within the CE abolishes the reporter activity (Table 1; L’Honore et al. 2010), indicating PITX2 binding to the CE is required for limb expression. In contrast, myotomal expression of *Myod* does not require *Pitx2*, but in *Pitx2*;*Myf5(Mrf4)* mutants almost all myotomal *Myod* expression is absent, suggesting redundancy between these factors (L’Honore et al. 2010). In support of a direct role in regulating *MyoD* expression in somites, chromatin immunoprecipitation experiments show PITX2 binding to the CE, DRR and just upstream of the PRR in mouse embryonic myotome (Table 1; Fig. 2; L’Honore et al. 2010). In the head conditional inactivation of *Pitx2* in the extraocular eye muscle precursors results in downregulation of *Myod* expression. ChIP of PITX2 in C2C12 cells and in an extraocular eye muscle derived cell line indicates that PITX2 binds the PRR but not the CE (the DRR was not tested; Table 1; Fig. 2; Zacharias et al. 2011). These data indicate that *Myod* expression is regulated through PITX2 binding a different combination of regulatory regions in the limbs (CE) compared to the myotome (CE, DRR, upstream of the PRR) and extraocular eye muscles (PRR), although how PITX2 discriminates is currently unknown.

### MUSCULIN and TCF21

The bHLH transcription factors, MUSCULIN (MSC; also known as MYOR) and TCF21 (CAPSULIN) are together required for formation of some first branchial arch-derived skeletal muscles, where they are transiently expressed (Lu et al. 2002). *Myod* expression is absent in these cells in *Msc*/*Tcf21* mutants and both factors bind the PRR and DRR (but not the CE) in E10.5 branchial arch cells, suggesting they directly regulate *Myod* expression (Table 1; Fig. 2; Moncaut et al. 2012).

### GLI2

Sonic hedgehog (Shh) signalling, which is mediated by the activating transcription factors GLI1 and GLI2, plays a role in regulating *Myod* expression both in the embryo and in regenerating muscle. Shh is required for expression of *Myod* in the epaxial myotome and limb and for initiation of *Myod* expression in the limb (Borycki et al. 1999; Hu et al. 2012). It is also required for the upregulation of *Myod* in activated satellite cells (Straface et al. 2009). In a cell culture model in which P19 cells are differentiated to myotubes, GLI2 activity was found to regulate *Myod* expression, possibly through GLI2 binding to sites ~  35 kb upstream of the CE and ~ 1 kb upstream of the *MyoD* TSS (Table 1; Voronova et al. 2013), raising the possibility that GLI2 directly regulates *Myod* expression in vivo.

### SRF, MEF2 and cofactors

Serum response factor (SRF), a transcription factor of the MADS box family, plays a role in skeletal, cardiac and smooth muscle formation (reviewed in Coletti et al. 2016). In skeletal muscle it is not required for embryonic *Myod* expression and myogenesis, but plays a role in adult muscle homeostasis. Members of the Myocardin family of transcription factors, including MRTF and MASTR, act as co-factors for SRF and MEF2 factors, also MADS box family members, respectively, and consequently also play important roles in muscle formation (Creemers et al. 2006; Olson and Nordheim 2010). MASTR and MRTF-A are up-regulated in satellite cells in response to injury, and are required for differentiation of satellite cells during growth and regeneration in the adult (Mokalled et al. 2012). Consistent with the DRR driving expression of *Myod* in adult muscle, all these factors bind the DRR (Table 1; Fig. 2). SRF binds to a divergent CArG box in the DRR, in complexes that include YY1, a regulator of enhancer–promoter interactions, CBP, a transcriptional coactivator, C/EBP family factors and MRTF-A (Table 1; Fig. 2; L’Honore et al. 2003, 2007; Mokalled et al. 2012; Weintraub et al. 2017). This CArG box is required for expression of a DRR-reporter construct in differentiating myoblasts in culture and in activated satellite cells after muscle lesion in vivo (L’Honore et al. 2003). The divergent CArG box is a combined MEF/SRF binding site and is also bound by a complex of MASTR and MEF2 family factors in myoblasts (Table 1; Fig. 2; L’Honore et al. 2007). It appears that a switch occurs as cells differentiate, such that while SRF binding initiates *Myod* expression, as cells differentiate MEF2 binding replaces SRF and maintains expression (L’Honore et al. 2007).

### CLOCK and BMAL1

In adult skeletal muscle, *Myod* mRNA exhibits circadian rhythm which is abolished in mice mutant for *Clock* or *Bmal1*, two bHLH-PAS domain transcription factors that heterodimerize to positively regulate the circadian clock through binding E-boxes in target gene loci. The CE is required to regulate periodic *Myod* expression in vivo, and both CLOCK and BMAL1 bind the CE in muscle (Andrews et al. 2010; Zhang et al. 2012). This binding appears to be via a conserved non-canonical E-box in the CE (Fig. 1b; Table 1; Zhang et al. 2012), and consistent with the CE regulating periodic activation of *Myod* transcription, CLOCK and BMAL1 binding to the CE in cultured cells is most enriched when *Myod* transcripts are at their highest (Andrews et al. 2010).

## Transcription factor binding to *Myod* cis-regulatory elements: repressors

*Myod* expression must also be repressed in a controlled temporal and spatial fashion. Reporter assays show that the DRR drives reporter gene expression in non-myogenic cells, suggesting that repressor sequences may be found outside the DRR region (Asakura et al. 1995). Some linker-scan mutations in the CE also result in ectopic expression, which suggests the CE contains regions that bind repressors (Kucharczuk et al. 1999). The evidence for repressor binding in the *Myod* upstream region is summarised below.

### SIM2

The bHLH-PAS domain transcription factor, *Sim2* is expressed in muscle progenitors shortly after they have migrated into the limb and before *Myod* expression is upregulated (Coumailleau and Duprez 2009; Havis et al. 2012). In *Sim2* mutants *Myod* expression in the limb is upregulated compared to wild type levels (Havis et al. 2012). Sim2 appears to prevent entry into the myogenic program through repression of *Myod* expression, since it binds to the CE in embryonic mouse limb buds and represses *Myod* transcription when overexpressed in primary limb bud myoblasts (Table 1; Fig. 2; Havis et al. 2012).

### DELTEX2

*Deltex2*, which encodes an E3 ubiquitin ligase, is expressed during regeneration in adult muscle in myogenic progenitor cells and inhibits myogenic differentiation (Luo et al. 2017). In cell differentiation assays, exogenously expressed DELTEX2 is able to bind the DRR and PRR, but not the CE (Table 1; Fig. 2), and leads to an enrichment of dimethyl lysine 9 of histone H3 (H3K9me2), a repressive chromatin mark, at the *Myod* TSS. This is likely to be through inhibiting the activity of JMJD1C, a lysine demethylase (Luo et al. 2017).

### C/EBP homology protein

C/EBP homology protein (CHOP, also known as DDIT3) is expressed in quiescent satellite cells in vivo and is transiently induced during myoblast differentiation in vitro (Alter and Bengal 2011; Fukada et al. 2007). Knockdown of CHOP in C2C12 cells results in premature expression of myogenic genes, and conversely overexpression of CHOP results in inhibition of the myogenic program and reduced nuclei numbers in myotubes that do form (Alter and Bengal 2011). CHOP seems to act by binding at approximately 3 kb upstream of the *Myod* TSS and repressing expression of *Myod* (Table 1; Fig. 2). Interestingly, this binding is associated with a decrease in Histone H4 acetylation (an active histone mark) near the DRR and may indicate CHOP acts through regulating the activity of a histone deacetylase (Table 1). Further evidence for this comes from experiments in 293 T cells where CHOP was shown to interact physically with Histone Deacetylase 1 (HDAC1; Alter and Bengal 2011).

## Epigenetic regulation of *Myod* regulatory regions

Whilst transcription factors direct the activation of gene expression, DNA must be accessible for them to bind. The accessibility of enhancer and promoter sequences to transcription factor binding is controlled through multiple epigenetic mechanisms including DNA methylation, histone modification and interactions with non-coding RNAs, all of which are at play in *Myod* regulation.

### Methylation

DNA methylation at cytosine (C) residues within CpG dinucleotides is generally associated with repression of gene expression. It has long been known that treating a mouse embryonic fibroblast cell line (CHC3H 10T1/2) with 5-azacytidine, which inhibits DNA methylation, can convert these cells to skeletal muscle, suggesting that DNA methylation is important in maintaining repression of myogenic genes in non-muscle cells (Constantinides et al. 1977). Indeed, DNA hypomethylation is associated with active enhancers (e.g. Blattler et al. 2014; Hon et al. 2013). This is seen at the CE, where studies in adult tissues and cell culture show that myoblasts and myotubes have low levels of methylation at the CE, while non-muscle tissues have higher levels (Brunk et al. 1996; Carrio et al. 2016; Ehrlich et al. 2016). Consistent with this, in the mouse embryo the presomitic mesoderm shows higher levels of methylation at the CE compared to the somites, with demethylation of CE preceding *Myod* expression (Brunk et al. 1996). However methylation of the CE may not be sufficient for repression of *Myod* expression, since a demethylated CE transgene is not activated precociously or ectopically, suggesting other mechanisms may also be responsible for active repression of *Myod* expression (Brunk et al. 1996).

### Nucelosomes and histone modification

In eukaryotes, DNA is wrapped around a core complex of histone proteins, the nucleosome, helping to package long DNA molecules into the nucleus but reducing the accessibility of transcriptional machinery to the DNA. Modification, such as acetylation and methylation, of amino acids in the N-terminal tails of histones alter how tightly the DNA interacts with the nucleosome and thereby regulates accessibility of transcription factor binding. As such nucleosome position and histone modification at enhancers and promoters are important regulators of gene expression. For instance, genome-wide studies using chromatin immunoprecipitation of histones have shown active promoters and enhancers often contain nucleosome-depleted regions, while nucleosomes downstream of promoters are associated with high levels of Histone H3 lysine 4 tri methylation (H3K4me3), and nucleosomes at enhancer regions are preferentially marked by high levels of H3K4me1 (Bernstein et al. 2006; Heintzman et al. 2007, 2009; Rada-Iglesias et al. 2011). The presence of H3K27ac marks promoters and enhancers as transcriptionally active, while H3K27me3, H3K9me2/3 and H4K20me3 are associated with repressed genes (Creyghton et al. 2010; Heintzman et al. 2007, 2009; Rada-Iglesias et al. 2011; Wang et al. 2008; Young et al. 2011). Acetylation of lysine residues in the Histone H4 tail are also associated with transcriptional activation (Wang et al. 2008).

These associations are reflected in studies of histone modification in muscle and non-muscle cells. Cell culture experiments show that myoblasts and myotubes both have active histone marks at the *Myod* promoter, DRR and CE, as well as other regions upstream of these, as does skeletal muscle, while non-muscle tissues have repressive marks in the same region upstream of the *Myod* gene (Ehrlich et al. 2016). For instance one study in a Rhabdomyosarcoma cell line, has shown that both the CE and the *Myod* TSS region are nucleosome free, and are associated with high levels of H3K4me1 and H3K4me3 respectively, while in colorectal cancer cells, where *Myod* is not expressed, both the CE and promoter contain nucleosomes (Table 2; Taberlay et al. 2011).

**Table 2:** Histone marks identified at the *Myod* proximal regulatory region (PRR), distal regulatory region (DRR) and core enhancer

Region	Histone	Type of mark	Binding shown by	Comments	References
PRR	H3K4me3	Active	ChIP-qPCR in Rhabdomyosarcoma cells		Taberlay et al. (2011)
	H3K27ac	Active	ChIP-qPCR in C2C12 cells	Levels increase as cells differentiate	Hamed et al. (2013)
	H4Kac	Active	ChIP-PCR in mouse embryo limbs	Lower levels than at CE	Havis et al. (2012)
	H3K9ac	Active	ChIP-qPCR in C2C12 cells	Levels increase as cells differentiate	Yang et al. (2011) and Hamed et al. (2013)
	H3.3	Active	ChIP-qPCR in C2C12 cells	Lower levels than CE. Levels increase as cells differentiate	Yang et al. (2011)
DRR	H3K4me3	Active	ChIP-qPCR in C2C12 cells	Levels increase as cells differentiate	Yang et al. (2011)
	H3K27ac	Active	ChIP-qPCR in C2C12 cells	Levels increase as cells differentiate	Hamed et al. (2013)
	H3K9ac	Active	ChIP-qPCR in C2C12 cells	Levels increase as cells differentiate	Yang et al. (2011) and Hamed et al. (2013)
	H3.3	Active	ChIP-qPCR in C2C12 cells.	Lower levels than CE. Levels increase as cells differentiate	Yang et al. (2011)
	H3K20me2	Repressive	ChIP-qPCR in satellite cells isolated from mouse muscle	Levels decrease in Suv4-20h1 knockout cells	Boonsanay et al. (2016)
	H3K9me2	Repressive	ChIP-qPCR in C2C12 cells		Luo et al. (2017)
CE	H3K4me3	Active	ChIP-qPCR in C2C12 cells	Levels increase as cells differentiate	Scionti et al. (2017) and Yang et al. (2011)
	H3K27ac	Active	ChIP-qPCR in and differentiated C2C12 cells	Levels increase greatly as cells differentiate	Hamed et al. (2013)
	H4Kac	Active	ChIP-PCR in mouse embryo limbs	Higher levels than at PRR	Havis et al. (2012)
	H3K4me1	Enhancer	ChIP-qPCR in Rhabdomyosarcoma cells		Taberlay et al. (2011)
	H3K9ac	Active	ChIP-qPCR in C2C12 cells	Levels increase as cells differentiate. Levels decrease when MSX1 expressed	Yang et al. (2011), Hamed et al. (2013) and Lee et al. (2004)
	H3.3	Active	ChIP-qPCR in C2C12	Higher levels than DRR and PRR. Levels increase as cells differentiate	Yang et al. (2011)
	H3K9me2	Repressive	ChIP-qPCR in C2C12	Levels increase when MSX1 expressed.	Lee et al. (2004)
	HB1	Repressive	ChIP-qPCR in C2C12	Levels increase when MSX1 expressed	Lee et al. (2004)

Enhancers are also often associated with P300, a transcriptional coactivator that contains a histone acetyltransferease (HAT) domain (Visel et al. 2009). P300 binding at the CE, DRR and PRR is found to increase as myoblast cells differentiate towards myotubes in C2 cell culture, with a corresponding increase in H3K27ac and H3K9ac (transcription elongation) levels (Table 2; Gates et al. 2017; Hamed et al. 2013). Whilst mouse mutants carrying an allele with reduced P300 HAT activity exhibit reduced *Myod* expression (Roth et al. 2003), suggesting that acetylation of histones at *Myod* regulatory regions is required for robust expression.

In addition to modification of core histones, variant histone isoforms, which have particular regulatory functions, are differentially incorporated into nucleosomes. For instance the Histone H3 variant, H3.3, is associated with active genes and enhancers (Ahmad and Henikoff 2002; Jin et al. 2009). At the *Myod* locus a study in C2 cells has shown that levels of H3.3 increase at the CE, DRR and PRR as myoblasts differentiate, and when levels of H3.3 are reduced at these regulatory regions through inhibiting the activity of the H3/H4 histone chaperone, Histone regulator A (HIRA), *Myod* transcription is reduced (Fig. 2; Yang et al. 2011), suggesting that transcription of *Myod* is facilitated by integration of Histone H3.3.

Few studies have looked at histone binding at the *Myod* locus in vivo, however Havis et al. (2012) showed that acetylated Histone H4 is present at the CE and PRR but not at the DRR in mouse embryos (Table 2). Interestingly, this reflects the situation seen in transgenic mice where the CE drives limb bud expression but the DRR does not (Asakura et al. 1995; Chen et al. 2001; Goldhamer et al. 1995).

### DNA looping

In active chromatin, loops of DNA are formed that bring the promoter and enhancer close together for activation of gene expression. The cohesion complex, which includes the RAD21 subunit, mediates this looping and thus many enhancers and promoters are associated with RAD21 binding. This is true of the *Myod* CE and promoter in Rhabdomyosarcoma cells, in which *Myod* is highly expressed, which are both associated with RAD21 binding (Fig. 2; Taberlay et al. 2011).

### Heterochromatin

In quiescent satellite cells levels of heterochromatin, which represses gene activation, are high and decrease as cells are activated and enter the myogenic pathway (Hawke and Garry 2001). In proliferating C2 cells, in which *Myod* expression is low, the CE is associated with high levels of H3K9me2/3 (Scionti et al. 2017), while in satellite cells isolated from adult mouse muscle the DRR is associated with high levels of H3K20me2, a substrate for further methylation to H3K20me3, which decrease as cells differentiate (Table 2; Boonsanay et al. 2016). This repression of *Myod* via deposition of H3K20me2 appears to be mediated by SUV4-20H1, a dimethyltransferase, since in satellite cells mutant for *Suv4*-*20h1*, *Myod* transcription increases while H3K20me2 is reduced and H3K4me3 is increased at the DRR (Table 2; Boonsanay et al. 2016).

Unlike the other histones, H1 sits atop the nucleosome, keeping the DNA wrapped around the core histone complex, and thus also plays an important role in regulating accessibility to the DNA (Izzo and Schneider 2016). During myogenesis, integration of the Histone H1b variant at the CE, mediated by binding of MSH Homeobox 1 (MSX1) and Y-box binding protein 1 (YB1) at the CE, is also associated with increased H3K9 methylation (Tables 1, 2; Lee et al. 2004; Song and Lee 2010). This is consistent with the role of MSX1 in repression of muscle differentiation in the hypaxial DM and migrating limb muscle progenitor cells in the mouse embryo (Fig. 2; Houzelstein et al. 1999).

### Enhancer RNAs

Another method of regulating accessibility of chromatin at the *Myod* locus is via an enhancer RNA that is transcribed from the CE (*CEeRNA*) (Mousavi et al. 2013; Scionti et al. 2017). Enhancer RNAs are a relatively recently discovered class of non-coding RNAs identified from whole genome studies. They are transcribed from active enhancers and appear to have important roles in regulating transcription (reviewed in Liu 2017). The mechanism by which the *CEeRNA* regulates *Myod* expression is still being investigated, but it seems to promote accessibility of the *Myod* promoter, since if it is depleted via siRNA then chromatin accessibility, as measured by DNAse1 sensitivity, is reduced, together with reduced *Myod* transcription levels (Mousavi et al. 2013). Expression of the *CEeRNA* during differentiation in C2 cells is promoted by binding of Lysine Specific Demethylase 1 (LSD1) to the CE (Fig. 1), whilst in mouse embryos a conditional knockout of *Lsd1* in muscle progenitor cells results in a strong reduction of *CEeRNA* expression in the limb (Scionti et al. 2017), indicating *Lsd1* is involved in *CEeRNA* expression in the embryo. Interestingly, this *Lsd1* conditional knockout also results in a strong reduction of initial *Myod* expression in the limbs and a mild reduction in somites, which recovers later, reminiscent of the *Myod* expression seen when CE is deleted in vivo (Chen and Goldhamer 2004). This suggests transcription of the *CEeRNA* is also required for CE activity in driving the initiation of *Myod* expression.

### Super-enhancers

Super enhancers are large regions of the genome that are characterised by high levels transcription factor occupancy, H3K27ac marks, mediator complex, and transcription of multiple enhancer RNAs (Khan et al. 2018). Interestingly, a region extending approximately 40–50 kb upstream of *Myod*, in both mouse and human locus, has been described that has characteristics of a super-enhancer (Ehrlich et al. 2016; Mousavi et al. 2013), which further implicates a larger region beyond 24 kb upstream of the *Myod* TSS in expression regulation (Fig. 1). Interestingly PAX3 has been shown to promote chromatin accessibility, and may act to do so in a region from the *Myod* TSS up to approximately 40 kb upstream, since in mouse embryoid bodies induced to express *Pax3*, both binding of PAX3 and accessibility of chromatin increases in this region during skeletal muscle differentiation (Magli et al. 2019).

## Conclusion

At first glance the *Myod* upstream region, with two main enhancer elements may seem uncomplicated. However, this belies a complex regulatory landscape where tissue and temporal specificity are merged, in the most part, into two these elements. This is in contrast to *Myf5* regulation, which has multiple enhancer regions located in a region 140 kb upstream of its TSS that drive distinct expression patterns in the somite, limb and head, and *Myog* which has just one 133 bp regulatory region located immediately upstream of its TSS (reviewed in Carvajal and Rigby 2010). While we have knowledge of many (but, undoubtedly, nowhere near all) of the transcription factors that bind *Myod* regulatory elements and the histone marks that decorate the region, we are far from understanding the details of how they interact with each other to bring about the precise spatial and temporal expression of *Myod*. For instance, while mutant studies can show that different transcription factors compensate for each other, what is their relative contribution in the wildtype situation? Similarly, what is the relative contribution of the CE and DRR and (how) do these regions communicate with each other to drive precise *Myod* expression? What is the relationship of transcription factor binding with epigenetic marks? Many studies have shown a correlation between transcription factor binding and epigenetic marks, but which comes first and interactions between all of them is yet to be fully explored. Non-coding RNAs, including the recent discovery of enhancer RNAs, have added another level of regulation that must be integrated into our picture of how all these different factors interact with each other to regulate *Myod* transcription.
